# The effects of plantar-flexor static stretching on perturbation recovery in the elderly

**DOI:** 10.1186/1757-1146-7-S1-A96

**Published:** 2014-04-08

**Authors:** Seong-gil Kim, Goonchang Yuk, Hwangbo Gak

**Affiliations:** 1 Department of Physical Therapy, College of Rehabilitation Science, Daegu University, Jilyang, Gyeongsan-si, Kyeongbuk, 712-714, Republic of Korea; 2 Department of Physical Therapy, Yeungnam University Hospital, 170 Hyeonchung-ro, Namgu, Daegu 705-703, Republic of Korea

## Background

It is important to improve the routine ADL(activities of daily living) in the elderly and then diverse and various therapeutic interventions or exercises are applied for the therapy. Generally, to increase the efficiency of the exercise and prevent the injury, the stretching is commonly used [1]. Indeed, there are many case that the elderly complain of the difficulties to control the balance after the stretching [2,3]. However, previous studies about the effects of stretching after or during the stretching have focused mainly on the histological or neurological changes and there are few studies that focused on the temporary balance control in the elderly [4,5]. Thus, the purpose of this study was to investigate the perturbation recovery of five minutes of plantar-flexor static stretching (PSS) in the elderly.

## Materials and methods

Thirty-one participants aged over 65 years performed 5 min-PSS in the form of wedge board standing. The sway length of each subject’s COM (center of mass) was measured to examine the subject’s static balance. It was measured for one minute in quiet standing with the eyes closed. Sway length was measured for 1 minute which was divided in three 20-second-sections before and after stretching.

## Results

The result showed significant decreases in sway length before stretching between 0-20s and 21-40s, 0-20s and 41-60s separately. However, the results between 21-40s and 41-60s did not show any significant changes.The result showed significant decreases in sway length after stretching between 0-20s and 41-60s, 21-40s and 41-60s. However, the results between 0-20s and 21-40s did not show any significant changes (Table 1).

**Table 1:** Comparison of sway length standing before and after 5 minutes plantar-flexor static stretch.

Condition	0-20sec	21-40sec	41-60sec	p
Pre-stretch	14.00±5.18	11.90±4.05	11.78±5.21	0.00^ac^

Post-stretch	15.87±6.14	15.32±6.13	13.75±5.41	0.02^bc^

*p<.05 (Mean±SD)

^a^ = 0-20sec * 21-40sec, ^b^ = 21-40sec * 41-60sec, ^c^ = 0-20sec * 41-60sec

## Conclusion

**Figure 1:**
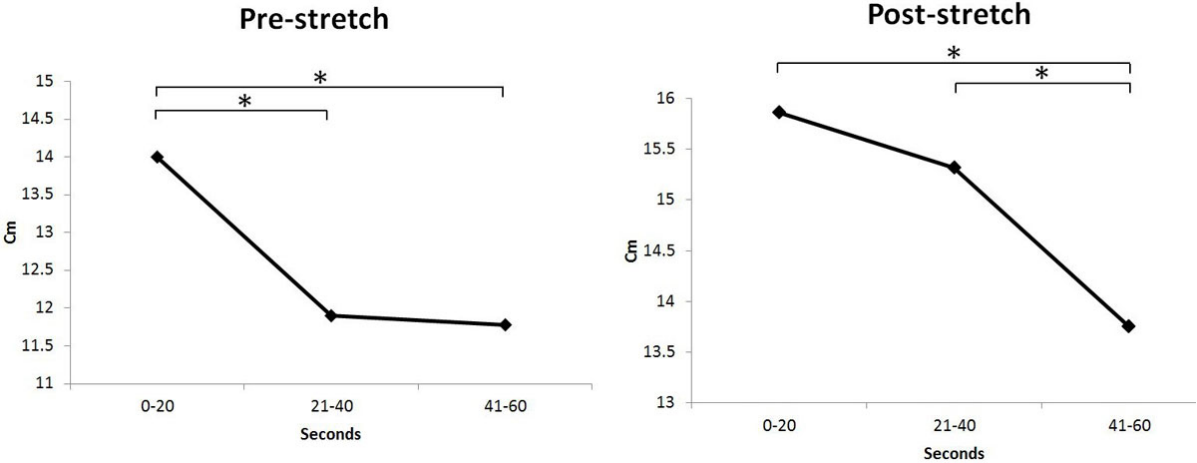
Comparison of sway length changes over the sections of 0-20, 21-40, 41-60s before and after stretch. *p<.05 (Mean±SD).

Stabilization time of sway length became stable from 21s before stretching with the eyes closed, but unstable duration lasted to 40s after stretching, and then sway length was started to decrease from that time(Figure 1). These results suggest that the elderly subjects temporarily experienced difficulties in maintaining balance immediately after the PSS. Therefore, to prevent falls and perform exercises in a safe way, it is recommended to allow patients to rest after performing PSS.

## Trial registration

Current Controlled Trials ISCRTN73824458.
